# Sex Differences in Associations Between Diet and Metabolic Health in Older Adults: The Roles of Vegetable Protein and Alcohol Intake

**DOI:** 10.3390/nu17213460

**Published:** 2025-11-02

**Authors:** Kayla R. Anderson, Philip A. Kern, Allison L. Steele, Brooke D. Lancaster, Madison Bee, Alyaa M. Zagzoog, Stacey A. Slone, Douglas E. Long, Jean L. Fry

**Affiliations:** 1 Center for Muscle Biology, College of Health Sciences, University of Kentucky, Lexington, KY 40536, USAbrookelancasterrdn@gmail.com (B.D.L.); alyaa.zagzoog@nbu.edu.sa (A.M.Z.); delong2@uky.edu (D.E.L.); 2 Department of Internal Medicine, College of Medicine, University of Kentucky Division of Endocrinology, Lexington, KY 40536, USA; 3 Department of Community Health, Faculty of Applied Medical Sciences, Northern Border University, Arar 73213, Saudi Arabia; 4 Dr. Bing Zhang Department of Statistics, College of Arts and Sciences, University of Kentucky, Lexington, KY 40536, USA; stacey.slone@uky.edu

**Keywords:** sex-based differences, insulin sensitivity, plant-based diet, body composition, alcohol intake, metabolic health

## Abstract

**Background/Objective:** Aging is associated with a decline in metabolic health, including impaired glucose regulation. Both diet and biological sex impact metabolic health, yet sexual heterogeneity in diet response is understudied. We report on exploratory analyses of sex-specific associations between diet and insulin sensitivity, insulin resistance, and android and intermuscular fat composition in older adults. **Methods:** This secondary analysis uses baseline data from a previously completed clinical trial (n = 96), the MASTERS study. An oral glucose tolerance test (OGTT) was used to calculate insulin resistance and insulin sensitivity as measures of metabolic function, while dual-energy x-ray absorptiometry and computed tomography were used to assess body composition. Univariate analyses were used to identify sex-specific associations between metabolic health and single nutrients, as well as other dietary components. Feasible solutions algorithm (FSA) modeling was employed to identify food groups that were most associated with insulin sensitivity. **Results:** In men, greater intakes of vegetable protein (*p* < 0.0001) and whole grains (*p* = 0.001) were associated with higher insulin sensitivity, while refined grains (*p* = 0.003) and conjugated linoleic acids (*p* < 0.001) were negatively associated. In women, insulin sensitivity was positively associated with alcohol (*p* < 0.001) and xylitol (*p* = 0.007). FSA modeling identified whole grains, nuts, and seeds as food groups that predicted higher insulin sensitivity in men, while alcohol remained the strongest predictor in women. **Conclusions:** Men showed higher insulin sensitivity with plant-based diets, while alcohol intake was the dietary factor most associated with insulin sensitivity in women. The findings of these exploratory analyses support the need for sex-specific clinical trials and dietary guidance for aging populations.

## 1. Introduction

Aging is a complex biological process associated with the decreased metabolic health and functional abilities of older adults. These declines are influenced by genetic, environmental, and lifestyle factors. Among these, diet plays a critical role in promoting metabolic health and longevity [1]. A well-balanced diet rich in certain nutrients, including essential micronutrients, antioxidants, soluble fiber, and protein, can mitigate chronic disease risks, promote physical function, and improve overall quality of life [2,3]. Furthermore, the adoption of specific dietary patterns, such as the Mediterranean diet, have been associated with successful aging outcomes [4]. However, some evidence indicates that diet affects cardiometabolic outcomes differently in men and women [5,6,7].

Ample evidence demonstrates that men and women metabolize nutrients differently. For example, following a high-fat meal, pre-menopausal women more efficiently oxidize fat in the liver when compared with age-matched men [8]. While sex-specific dietary recommendations may support the maintenance of optimal metabolic health in older adults, the health implications of these differences in metabolism and their interaction with diet remain unclear.

Glucose metabolism and insulin sensitivity are key aspects of cardiometabolic health affected by dietary choices. Aging-associated changes in glucose regulation, impaired glucose tolerance, and impaired fasting glucose increase the risk of type 2 diabetes and cardiovascular disease, and older adults are often diagnosed with these conditions [9,10]. Insulin resistance, hyperinsulinemia, and excess activation of the insulin signaling pathway have been identified as key drivers of aging-related chronic inflammation [11], which is often called inflammaging [12]. Moreover, hyperinsulinemia has been directly linked with accelerated aging processes [13]. Consequently, strategies to enhance insulin sensitivity, such as caloric restriction, intermittent fasting, and pharmacological interventions like metformin, have shown promise for reducing the pace of aging [14], oxidative stress [15], and the activity of signaling pathways downstream of insulin, for example the mechanistic target of rapamycin (mTOR) [16]. Dietary interventions that bolster dietary quality and reduce circulating insulin are a viable approach for mitigating inflammaging and enhancing metabolic health in older adults.

The relationship between diet, nutrients, and glucose metabolism has been extensively studied. However, there is a knowledge gap concerning sex-specific associations between dietary components and insulin sensitivity, especially in the context of human aging. Therefore, the primary purpose of these exploratory secondary analyses is to identify nutrients and food groups that are associated with insulin sensitivity in otherwise healthy older adults. We hypothesized that the nutrients and food groups associated with insulin sensitivity would differ between men and women, supporting the need for clinical trials to define sex-specific dietary guidelines for successful aging and metabolic health.

## 2. Materials and Methods

### 2.1. Participants and Parent Study

This study is a secondary analysis of the Metformin to Augment Strength Training Effective Response in Seniors (MASTERS) [17,18], which was a clinical trial in generally healthy older adults aimed at examining muscle mass and function in response to progressive resistance exercise training with and without the use of metformin [17,18]. Participants (n = 96) were recruited from the community at the University of Kentucky (UK) and the University of Alabama at Birmingham (UAB). The MASTERS study included older adults (median age approximately 69 years) and had equal enrollment of males and females. BMI ranged from approximately 19 to 34 kg/m^2^. Although this BMI range exceeds the “normal” classification for BMI, some evidence indicates that a healthy BMI threshold up to 30 kg/m^2^ in older adults may support better health outcomes [19]. Additionally, BMI may be a less reliable indicator of adiposity in older adults when compared with younger people [20]. Participants exhibited moderate to high levels of physical function as assessed by the Short Physical Performance Battery (SPPB). The participants were not receiving hormone replacement therapy. From this parent study, we utilized data from all participants who had both baseline dietary data, as well as baseline data from the oral glucose tolerance test (OGTT; n = 89), dual-energy x-ray absorptiometry (DXA; n = 96), or computed tomography (CT; n = 96). The dietary and OGTT data have not been published previously, excepting broad summary statistics included in the article reporting the original clinical trial [17,18]. All participants provided informed consent, and the study was approved by the University of Kentucky Institutional Review Board (#47128).

### 2.2. Diet and Dietary Supplement Data Collection

Participants’ diet was evaluated with a 4-day food record, which included three weekdays and one weekend day. Participants received written and verbal instructions on how to record all foods, beverages, and dietary supplements consumed, including portion sizes, preparation methods, and brand names when applicable. Participants were encouraged to use household measuring tools (e.g., measuring cups, spoons, food scales) and were provided with a portion size estimation guide.

### 2.3. Dietary Analysis

Data from the MASTERS study baseline food and dietary supplement record collection were analyzed using the Nutrition Data System for Research software (NDSR 2019 release, Nutrition Coordinating Center, University of Minnesota, Minneapolis, MN, USA). Entries from food records were manually entered into NDSR by trained research staff and reviewed by a registered dietitian. NDSR was used to generate estimates of energy intake (kcal), macronutrients (carbohydrates, protein, fat), fiber, vitamins, minerals, and specific bioactive compounds (e.g., phytoestrogens, fatty acids, and inositol compounds). All individual nutrients reported by NDSR were included in our univariate analyses, and dietary and supplemental sources of nutrients were merged prior to analysis. To account for variations in energy intake, energy-yielding components were analyzed as both absolute quantities and normalized per 1000 kcal and body weight. Additional variables were also created for nutrients that have recommendations in the US differing from the units in the output, for example the percentage of overall energy intake for added sugar.

Additionally, food group intake data (e.g., whole grains, refined grains, dairy, nuts, and seeds, processed and fresh meats) were calculated based on predefined categories in the NDSR system, which were merged into 25 combined food groups (Supplemental File S1) to reduce the number of variables in the multiple regression data analysis.

### 2.4. Insulin Sensitivity and Insulin Resistance Calculations

Participants completed a 75 g oral glucose tolerance test (OGTT) following an overnight fast of at least 8 h. At the beginning of the test (0 min), a baseline blood sample was collected to measure fasting glucose and insulin levels (n = 88) as previously described [17,18]. Participants then consumed a standardized 75 g glucose solution within 5 min, followed by blood sample collection at 30-, 60-, 90-, and 120 min post-ingestion. The Homeostatic Model of Insulin Resistance (HOMA-IR; n = 89) [21] and Matsuda Insulin Sensitivity Index (MAT-ISI; n = 88) [22] were used to estimate hepatic insulin resistance and whole-body insulin sensitivity, respectively.

### 2.5. Dual-Energy X-Ray Absorptiometry (DXA)

Body composition was assessed via DXA using a GE Lunar iDXA scanner (GE Healthcare, Chicago, IL, USA). Scans were performed with participants in a supine position using standard imaging protocols [18]. All scans were analyzed using GE Lunar software version 10.0. The specific outcome variable of interest was the percentage of android region fat, defined as the ratio of android region fat (grams) to overall total mass in the android region (both lean and fat grams).

### 2.6. Computed Tomography Scans

Intermuscular fat (IMF) was assessed using a single-slice CT scan at the mid-thigh, defined as the midpoint between the inguinal crease and the proximal border of the patella with the hip and knee flexed at approximately 90°. CT scans were performed on either a GE Discovery CT750 HD (UAB) or a Siemens Somatom Definition (Malvern, PA, USA) scanner. A 5 mm thick cross-sectional image of the right thigh was obtained using 100 mA with a scanning time of 3 s and a 512 × 512 matrix resolution. The scan was aligned to the predefined midpoint mark on each participant’s thigh as previously described [18]. Fat area was quantified using attenuation values from the CT scan images, analyzed with NIH ImageJ software (https://imagej.net/ij/, accessed on 28 October 2025). We used IMF area standardized to total fat area in the CT scan as a secondary outcome variable in our analyses.

### 2.7. Statistical Analyses

To complete an exploratory analysis of which of the 201 absolute and adjusted nutrient and 13 other health-related measures (e.g., BMI) significantly associated with the outcome measures, univariate regression models were run for each measure stratified by sex. To account for multiple comparisons in these univariate analyses, the significance threshold was set at *p* < 0.01. As a sensitivity analysis to address potential energy under-reporting, we repeated partial correlation analyses adjusted for body weight and total energy intake in the subset of participants (n = 77) whose reported energy intake met or exceeded estimated resting energy requirements calculated using the Mifflin–St Jeor equation [23]. Benjamini–Hochberg false discovery rate correction was applied to the *p*-values from these tests.

To assess the food group variables (absolute portions), candidate models, implemented for males and females separately, were generated using the feasible solutions algorithm (FSA) [24]. The FSA searches through a random selection process to find the two variables (and their two-way interaction) that locally maximizes the R^2^ of the regression line predicting the outcome. After reviewing the two-way covariate interaction models were selected by the FSA to confirm the model with the largest R^2^ for each outcome variable, the four analysis of variance models were then contrasted to demonstrate robustness. These models included the significant interaction, a model adding just baseline BMI, a model adding only minutes of weekly exercise, and a model including both covariates. ANOVA model assumptions were reviewed and confirmed including normality of the outcomes. The FSA analyses were run using the rFSA package in R, version 4.1.3 (R Foundation for Statistical Computing, Vienna, Austria) and model comparisons and a Benjamini–Hochberg adjustments were run using SAS 9.4 (SAS Institute Inc., Cary, NC, USA). Descriptive statistics, partial correlations, and t-tests comparing baseline demographic and health indicators and intakes of nutrients of interest between men and women were completed using IBM SPSS Statistics v.29.

## 3. Results

### 3.1. Participant Characteristics

Participants were older, generally healthy American adults. Women tended to be metabolically healthier than the men with higher Mat-ISI, lower fasting glucose, and lower HOMA-IR, on average (Table 1).

### 3.2. Dietary Intake in Older Men and Women

Dietary intake was evaluated among 47 males and 49 females. Significant sex differences were observed for energy intake, as males consumed more daily kilocalories in comparison to females, while females consumed more total fiber, which was driven by higher insoluble fiber intake (Table 2).

### 3.3. Sexual Dimorphism in Nutrient Association with Metabolic Health

#### 3.3.1. Insulin Sensitivity Assessed with Mat-ISI

**Figure 1:**
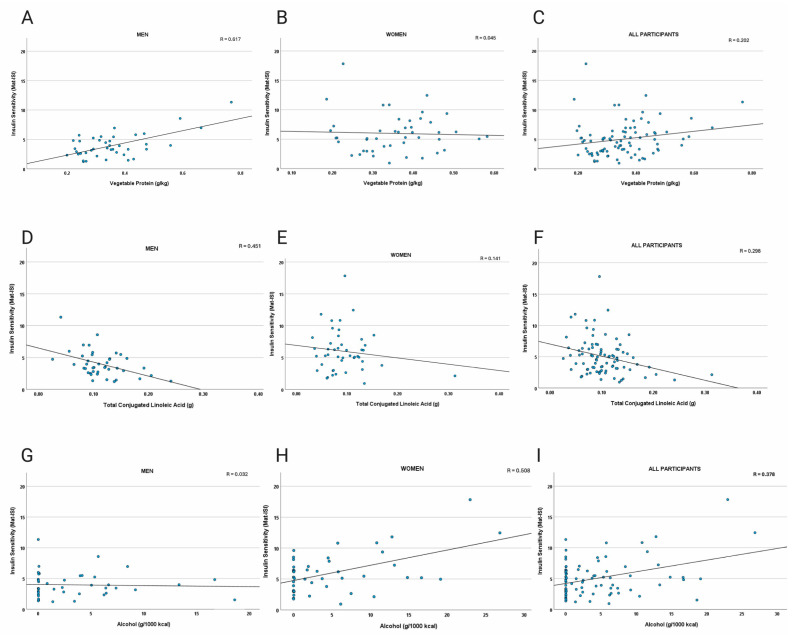
Scatter plots of dietary factors versus Matsuda Insulin Sensitivity Index (Mat-ISI). Panels depict vegetable protein intake in men (**A**), women (**B**), and all participants (**C**), with positive associations in men; total conjugated linolenic acid intake in men (**D**), women (**E**), and all participants (**F**), with negative correlations strongest in men; and alcohol intake in men (**G**), women (**H**), and all participants (**I**), with no relationship in men and positive correlations in women. Univariate analyses indicating significant associations are reported in Table 3 and Table 4).

In women, Mat-ISI was positively associated with intake of alcohol (g/1000 kcal; *p* < 0.001 total grams, *p* = 0.005) and xylitol (*p* = 0.007; Table 3), whereas BMI and body weight were inversely associated with insulin sensitivity (*p* < 0.001). In men, vegetable protein (*p* < 0.0001), whole grains (*p* = 0.001), inositol (*p* = 0.002), phytic acid (*p* = 0.002), vitamin E (*p* = 0.003), and oxalic acid (*p* = 0.003) were among the dietary variables most positively associated with Mat-ISI (Table 4). In addition to BMI and total body weight (*p* < 0.0001 and *p* = 0.0002, respectively), refined grain (*p* = 0.003) and conjugated linoleic acids (*p* = 0.003) were most negatively associated with Mat-ISI in men. There were no differences in intake of these food components and nutrients between the sexes (Table 2). Scatter plots of a subset of the data points are presented in Figure 1.

In the sensitivity analysis restricted to participants meeting estimated resting energy needs (n = 77), partial correlations adjusted for body weight and energy intake produced results consistent with the main analysis, though Benjamini–Hochberg FDR-adjusted *p*-values > 0.05 for alcohol and xylitol in women (Supplemental File S2).

#### 3.3.2. Insulin Resistance Assessed with HOMA-IR

Univariate analyses showed significant sex-specific associations between dietary intake and insulin resistance (HOMA-IR). In females, higher BMI and weight were positively associated with HOMA-IR (*p* < 0.0001), reinforcing the strong role of adiposity in insulin resistance. Analyses showed no significant associations between HOMA-IR and dietary components in women. In males, higher energy-adjusted trans-fat intake (*p* < 0.001), total solid fatty acids (*p* = 0.003), and trans-18:1 (trans-octadecenoic acid; *p* = 0.006) were positively associated with insulin resistance (*p* < 0.01), while vegetable protein was inversely associated (*p*< 0.003649) suggesting that processed and solid fats associate with fasted insulin resistance in men (Table 5).

#### 3.3.3. Android Fat

In women, total protein (<0.000001), calcium (*p* = 0.008) and self-reported number of alcoholic beverages per week (*p* = 0.008) were associated with a lower proportion of body fat from android depots (Table 6). For android fat percentage (android region fat adjusted for total android region mass), men consuming more vegetable protein (both weight- and energy-adjusted *p* < 0.001), phytic acid (*p* < 0.001), whole grains (*p* < 0.001), weight-adjusted total carbohydrate (*p* < 0.001), dietary fiber (*p* = 0.001), d-alpha tocopherol (i.e., natural vitamin E), and insoluble fiber (*p* = 0.002), were associated with lower relative abdominal fat, whereas intake of cis-9, trans-11 CLA and total CLA were associated with higher android fat percentage (*p* ≤ 0.004; Table 7).

#### 3.3.4. Intermuscular Leg Fat

Finally, intermuscular leg fat was inversely associated with soy isoflavones (glycitein and genistein) in women (*p* < 0.01), but no relationships were observed in men.

### 3.4. Modeling the Association of Food Groups with Metabolic Health

After identifying individual nutrients that are associated with metabolic health in each sex, we used multiple linear regression to model how intake of food groups is associated with the primary outcome of insulin sensitivity in both men and women while considering the potential impact of BMI and exercise on the outcome.

#### 3.4.1. Alcohol Intake Positively Associates with Insulin Sensitivity in Women

For females, Model 1 showed that consideration of food group intake explained significant variability in the Mat-ISI (F(3, 47) = 9.68, *p* < 0.0001, R^2^ =0.382; Table 8). Significant predictors included alcohol intake (β = 3.12, *p* < 0.001), which retained a similar effect in all models, and an interaction between alcohol and salty condiments (β = −2.58, *p* = 0.004). When BMI was added (Model 2), the model fit improved (F(4,46) = 12.66, *p* < 0.0001, R^2^ = 0.524). Adding only exercise (Model 3) weakened the overall model (F(4,46) = 7.11, *p* = 0.0002, R^2^ = 0.382), while the combined BMI and exercise model (Model 4) resulted in the best fit (F(5,45) = 9.91, *p* < 0.0001, R^2^ = 0.524). In Model 4, higher BMI was associated with lower insulin sensitivity (β = −0.42, *p* < 0.001), while exercise was not significantly predictive (*p* = 0.933). The results from all models indicate that alcohol intake is associated with higher insulin sensitivity in women, but salty condiments in the diet weaken the association. We speculate that overall dietary patterns that include foods where salty condiments are often used (e.g., fast foods) may diminish the positive association between alcohol and insulin sensitivity in women.

#### 3.4.2. Plant Foods Posivitively Associate with Insulin Sensitivity in Men

For males, the food group-only model (Model 1) for Mat-ISI was significant (F(3,38) = 10.22, *p* < 0.0001, R^2^ = 0.446; Table 9) and showed an interaction effect for intake of the nut and seed and whole grain groups, which were positively associated with Mat-ISI (β = 0.26, *p* = 0.002). Adding BMI alone (Model 2) retained the positive interaction effect between seed and nut and whole grain intake (β = 0.247, *p* = 0.002) and improved the model (F(4,37) = 12.72, *p* < 0.0001 R^2^ = 0.579). Exercise alone (Model 3) was also significant (F(4,37) = 9.19, *p* < 0.0001, R^2^ = 0.498), while the combined BMI and exercise model (Model 4) explained the most variance (F(5,36) = 11.06, *p* < 0.0001 R^2^ = 0.606). Combined, the data suggest that an overall dietary pattern including seeds and nuts, in combination with whole grains, is associated with higher insulin sensitivity in men. Seeds, nuts, and whole grains accounted for less than 50% of total vegetable protein in men, so the results of the food group modeling support that healthful sources of vegetable protein are associated with higher insulin sensitivity in men.

## 4. Discussion

This exploratory analysis examined sex-specific associations between dietary intake and markers of metabolic health in older adults. Key findings indicate stronger associations between diet and metabolic health in men, as well as a tendency for men to exhibit higher insulin sensitivity when consuming a healthy, plant-based diet. Men consuming higher amounts of vegetable protein, whole grains, and certain plant micronutrients and phytochemicals demonstrated lower levels of android fat and greater insulin sensitivity. Among women, moderate alcohol intake was associated with higher insulin sensitivity, while greater total protein from any source and calcium associated with less android fat.

### 4.1. Plant-Based Diets and Insulin Sensitivity

The positive association between plant-based diet components and insulin sensitivity in men builds on existing evidence supporting the metabolic benefits of plant-based diets [6,25]. Previous studies have demonstrated that higher intakes of whole grains, legumes, and plant-derived bioactive compounds are associated with improved glucose metabolism and lower risk of type 2 diabetes in men [26,27]. These findings align with limited previous research indicating that plant-based protein sources reduce risk of developing type 2 diabetes and CVD in men [5,28], whereas diets rich in overall protein, even from animal sources, are associated with lower risk of developing type 2 diabetes in women [5]. That said, our modeling of food groups confirmed that legumes and whole grains, the food groups providing substantial plant protein to the diet, were associated with insulin sensitivity in men. These results suggest that the positive association between grams of plant protein and insulin sensitivity are at least partially explained by the overall dietary pattern rather than plant protein alone. Previously reported sex differences in protein metabolism further underscore the need for individualized dietary recommendations based on biological sex [29,30,31,32].

### 4.2. Plant Phytochemicals

Our findings also reinforce a positive association between inositol and phytic acid, a storage form of inositol, in metabolic health. Inositol is a group of bioactive compounds abundant in whole grains, legumes, and nuts. Myoinositol (MI) plays a critical role in insulin signaling as a precursor in the synthesis of phosphatidylinositol 3,4,5-trisphosphate (PIP3), a key component of the insulin signal transduction pathway. Within skeletal muscle, MI is enzymatically converted into D-chiro-inositol (DCI), which enhances insulin signaling through mechanisms distinct from MI. Some evidence supports that supplementation with MI and DCI improves insulin sensitivity [33], particularly in men [33], suggesting that dietary inositol could be a promising adjuvant therapy in men with insulin resistance.

### 4.3. Animal-Derived Fats

Here CLA, particularly cis-9, trans-11, was negatively associated with insulin sensitivity in men. This isomer of CLA, is a product of ruminant animals and found in the diet in meat and dairy products. It is the naturally occurring isomer and accounts for most of the total CLA consumed in a habitual animal-based diet and has previously been shown to increase GLUT4 expression and decreased macrophage infiltration of white fat in male ob/ob C57BL-6 mice [34]; however, the other common CLA isomer, synthetic trans-10, cis-12, typically produced synthetically and consumed as an oral supplement, caused increases in insulin concentrations and insulin resistance in C57Bl/6J male mice [35] and decreases in insulin sensitivity in men [36].

Since men in our data set did not supplement with CLA, their intake of the trans-10, cis-12 was low. Therefore, we speculate that the inverse relationship between CLA intake and insulin sensitivity in men is due to dietary patterns emphasizing high-fat animal foods rather than the effects of the cis-9, trans-11 CLA isomer itself. Given the complexity of human nutrition, it is overly reductive to classify a single nutrient as “beneficial” or “harmful” without consideration of overall diet and other behaviors that affect health. Therefore, our findings should not be interpreted as evidence that older men should avoid foods containing cis-9,trans-11 CLA isomer (e.g., dairy, animal proteins). No associations between CLA and metabolic outcomes were observed in women, potentially due to lower intake levels which may have limited the ability to detect associations.

### 4.4. Alcohol in Women

Unlike men, insulin sensitivity in women was positively associated with alcohol and xylitol intake. Men and women consumed comparable amounts of alcohol in this study, averaging approximately 4 g per 1000 kcal each day, which is considered low, given that a standard alcohol “serving” like one typical 12-oz beer has approximately 14g of ethanol. Previous research has suggested that low levels of alcohol consumption may have cardiometabolic benefits, especially reduced risk of hypertension, particularly in women, when compared with men [37,38,39,40]. While human studies directly and prospectively investigating sexual dimorphism in metabolic responses to alcohol intake are limited, there is limited evidence that chronic alcohol intake results in lower glucose concentrations in female rats, but not male rats, even in the absence of acute alcohol ingestion [40]. Additionally, a meta-analysis in adults without diabetes showed a relationship between moderate alcohol intake and reduced HbA_1_c and fasting insulin among all participants, but lower insulin sensitivity in women only [40].

**Figure 2:**
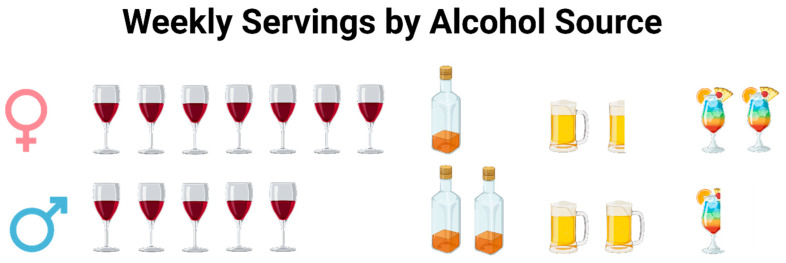
Weekly servings by alcohol source in women and men. Women (**top**) tended to consume more wine and cocktails, while men (**bottom**) tended to consume fewer cocktails and more liquor and beer.

Given that alcohol consumption had one of the strongest associations with insulin sensitivity in women, our findings support that there is potentially sexual dimorphism in response to low-moderate chronic alcohol ingestion. On the other hand, average alcohol intake was similar between the sexes, but the alcohol source varied (Figure 2). While both sexes drank more wine than any other alcoholic beverage, women consumed it to a greater extent. Similarly, xylitol, which was sourced primarily from wine and whole dark red and purple fruits in female participants’ diets, was positively associated with insulin sensitivity in our analyses. Since total xylitol intake was under a gram each day, even for the heaviest consumers, the association between these alcohol and insulin sensitivity may be more linked to the intake of dark fruits in various forms. Especially considering the lack of association between alcohol and insulin sensitivity when removing under reporters, the association between alcohol and insulin sensitivity must be interpreted with caution. First, alcohol and wine intake can co-occur with broader dietary patterns. For example, wine drinkers may also consume more fruit or polyphenol-rich foods. Second, we did not perform formal tests excluding abstainers or former drinkers because these data were not available. Finally, the alcohol × salty-condiments interaction should be interpreted cautiously because it may reflect an underlying dietary pattern rather than a biologic interaction between the two dietary variables.

Our findings should not be interpreted as a recommendation to increase alcohol intake, especially given the dose–response risk, but should reflect beverage context and dietary pattern rather than alcohol alone. The reported associations should be considered exploratory and hypothesis-generating. The modest relationship emphasizes the need for prospective studies to clarify alcoholic beverage specific mechanisms and effects in women.

### 4.5. Limitations

Several limitations should be considered when interpreting our findings. First, the study utilized a secondary analysis of baseline data from a clinical trial so the relationships identified are purely associative. We were limited by the sample size of the original study, and the modeling would have been more robust with a greater number of participants. Individuals with better insulin sensitivity may be more likely to follow a plant-based diet and participate in other healthy behaviors. Although the modeling did adjust for some potential confounders (i.e., BMI, exercise), other variables related to lifestyle and behavior factors may influence both dietary intake and metabolic outcomes. Additionally, all self-reported dietary intake may be subject to measurement error and recall bias. Other factors can affect the outcomes studied here, including socioeconomic status, smoking, comorbidities (Supplemental Table S1), and medications (Supplemental Table S2). The generalizability of our findings is limited because the sample was relatively homogeneous (older, generally healthy, predominantly Caucasian older adults), and results may not extend to younger, more ethnically or socioeconomically diverse, higher-comorbidity, or clinical populations.

Finally, although we report OGTT-derived indices (HOMA-IR as a fasting/hepatic proxy and the Matsuda index for whole-body insulin sensitivity), these surrogate measures do not fully capture age-related dysregulation of glucose metabolism. Aging is commonly accompanied by a reduced ability of the pancreas to compensate for declining insulin sensitivity, altered digestion and metabolism of carbohydrates (including altered gastric emptying and incretin responses), and greater glycemic variability [10]. These factors and others can influence metabolic outcomes. Follow-up studies could use other assessments like C-peptide from the OGTT, continuous glucose monitoring, and euglycemic-hyperinsulinemic clamp to clarify the relative contributions of β-cell function, liver glucose metabolism, and peripheral insulin sensitivity, and to examine how these processes interact with dietary exposures in older adults. Future research should include powered studies to confirm differential responses to diet in men and women and should define mechanisms underlying the sex-based differences in metabolic responses to diet.

## 5. Conclusions

Our analysis highlights significant sex-specific associations between diet and metabolic health in older adults. Our findings show that plant-based dietary patterns, particularly those rich in vegetable protein, whole grains, and inositol, are associated with higher insulin sensitivity in men, whereas alcohol and xylitol intake are associated with better insulin sensitivity in women. These results contribute to the growing body of evidence supporting the need for sex-specific dietary recommendations to promote metabolic health. Further research is needed to elucidate the mechanisms underlying these associations and to inform precision nutrition strategies for older adults.

## Figures and Tables

**Table 1:** Anthropometric and Metabolic Health Indicators.

Variables (n = 96)	Men (n = 47) Mean ± SD	Women (n = 49) Mean ± SD	*p*-Value ^1^
Age (years)	71.5 ± 5.6	69.2 ± 3.2	0.016
Weight (kg)	85.3 ± 10.8	68.6 ± 9.9	<0.001
Body Mass Index	27.3 ± 2.7	25.6 ± 3.4	0.006
Systolic BP ^2^ (mmHg)	129.7 ± 15.8	123.1 ± 14.4	0.036
Diastolic BP (mmHg)	75.0 ± 9.9	71.1 ± 10.1	0.060
Fasting glucose (mg/dL) *	99.4 ± 8.3	92.0 ± 12.3	0.002
Mat-ISI ^3^*	4.0 ± 2.0	6.0 ± 3.3	<0.001
HOMA-IR ^4^*	2.5 ± 1.5	1.6 ± 1.0	0.002
Android Region % Fat ^5^	40.0 ± 8.7	41.6 ± 10.4	0.382
Intermuscular Fat Ratio ^6^	0.24 ± 0.17	0.12 ± 0.04	<0.001

^1.^ Anthropometric and metabolic health indicators were compared using independent t-tests; ^2.^ Blood Pressure; ^3.^ Matsuda Insulin Sensitivity Index; ^4.^ Homeostatic Model of Insulin Resistance; ^5.^ Android region fat (grams) as a ratio of total mass in the android region (both lean and fat grams); ^6.^ Intermuscular fat area (cm^2^) as a ratio of all fat area identified on the computed tomography (CT) scan; * n = 89 for HOMA-IR and n = 88 for MAT-ISI.

**Table 2:** Nutrient intake in males and females.

Nutrient	Men (n = 47) Mean ± S.D.	Women (n = 49) Mean ± S.D.	*p*-Value
Total Energy (kcal)	1960 ± 471	1610 ± 421	<0.001
Energy (kcal/kg)	23.7 ± 5.6	23.9 ± 6.4	0.876
Total Fat (g/kg)	0.98 ± 0.29	0.98 ± 0.32	0.919
Saturated Fat (g/kg)	0.31 ± 0.09	0.31 ± 0.16	0.851
Total Omega-3 (g)	2.2 ± 1.1	2.0 ± 1.2	0.417
Total Protein (g/kg)	0.98 ± 0.19	0.98 ± 0.27	0.935
Animal Protein (g/kg)	0.63 ± 0.17	0.62 ± 0.25	0.866
Vegetable Protein (g/kg)	0.35 ± 0.12	0.35 ± 0.10	0.760
Total Carbohydrate (g/kg)	2.69 ± 0.84	2.74 ± 0.77	0.751
Total Dietary Fiber (g/1000 kcal)	11.2 ± 3.2	13.9 ± 3.6	<0.001
Soluble Dietary Fiber (g/1000 kcal)	4.3 ± 1.7	4.7 ± 1.7	0.174
Insoluble Fiber (g/1000 kcal)	6.8 ± 2.1	9.0 ± 2.4	<0.001
Whole Grains (oz/1000 kcal)	0.79 ± 0.62	0.92 ± 0.75	0.38
Refined Grains (oz/1000 kcal)	2.5 ± 1.0	2.1 ± 1.0	0.063
Total Alcohol (g)	7.2 ± 10.1	7.8 ± 10.9	0.787
Alcohol (g/1000 kcal)	3.9 ± 5.4	4.8 ± 6.6	0.477
Conjugated Linoleic Acid (g)	0.11 ± 0.04	0.09 ± 0.05	0.021
Vitamin E (α-Tocopherol) (mg)	11.6 ± 6.1	11.1 ± 5.5	0.660
Xylitol (g)	0.02 ± 0.01	0.02 ± 0.01	0.477
Inositol (g)	0.40 ± 0.27	0.37 ± 0.16	0.622
Phytic Acid (mg)	684 ± 326	677 ± 298	0.912
Oxalic Acid (mg)	247 ± 152	209 ± 113	0.167
Genistein (mg)	0.70 ± 1.63	0.95 ± 1.78	0.461
Glycitein (mg)	0.10 ± 0.25	0.14 ± 0.28	0.482
Eating Window (hours)	11.1 ± 1.1	11.1 ± 1.6	0.870

**Table 3:** Dietary Factors Associated with Insulin Sensitivity in Women.

Variables (n = 46)	Estimated Coefficient	*p*-Value ^1^
Alcohol (g/1000 kcal)	0.25	<0.0001
Alcohol (g)	0.1189	0.005113
Xylitol (g)	108.7164	0.006530

^1^ Univariate regression models identified dietary components associated with Matsuda Insulin Sensitivity Index with significance threshold *p* < 0.01.

**Table 4:** Dietary Factors Associated with Insulin Sensitivity in Men.

Variable (n = 43)	Estimated Coefficient	*p*-Value ^1^
Vegetable Protein (g/1000 kcal)	26.6332	0.000006
Vegetable Protein (g/kg)	10.4011	0.000017
Whole Grains (oz)	0.7872	0.001179
Inositol (g)	3.4844	0.001619
Phytic Acid (mg)	0.0030	0.001657
Refined Grains (oz/1000 kcal)	−1.0181	0.002691
Total CLA ^2^ (g)	−22.0141	0.002749
Vitamin E (α-Tocopherol) (mg)	0.2031	0.002813
CLA ^2^ cis-9, trans-11 (g)	−26.7475	0.002914
Oxalic Acid (mg)	0.0059	0.003470
Total Omega-3 Fatty Acids (g) Vegetable Protein (g)	0.8110	0.003544
RRR-α-Tocopherol (mg)	0.0920	0.003578
Insoluble Fiber (g/1000 kcal)	0.2288	0.003996
Whole grain (oz/1000 kcal)	0.4308	0.006289
Insoluble Fiber (g)	1.3734	0.007135
Animal protein (g)	0.1602	0.007196
Pectins (g)	−0.0544	0.007890
Refined Grains (oz)	0.5698	0.009153
	-0.3881	0.009958

^1.^ Univariate regression models were run to identify dietary factors associated with Matsuda Insulin Sensitivity Index with significance threshold *p* < 0.01. ^2.^ Conjugated Linoleic Acid.

**Table 5:** Dietary Factors Associated with Insulin Resistance in Men.

Variables (n = 43)	Estimated Coefficient	*p*-Value ^1^
Total Trans-Fatty Acids (g)	1.4864	0.000654
Solid Fats (g/1000 kcal)	0.1245	0.002973
Vegetable Protein (g/1000 kcal)	−13.276	0.003649
Trans-octadecenoic acid (g)	0.5952	0.005853
Total Trans Fatty Acids (g)	0.5401	0.006235

^1.^ Univariate regression models were run to identify dietary factors associated with HOMA-IR with significance threshold *p* < 0.01.

**Table 6:** Associations between dietary factors and android region fat percentage in women.

Diet Component (n = 49)	Estimated Coefficient	*p*-Value ^1^
Total Protein (g/kg)	−20.5690	<0.000001
Alcoholic drinks per week ^2^	−1.2946	0.008008
Calcium (mg)	−0.0067	0.008276

^1.^ Univariate regression models were run identify dietary factors associated with DXA Android fat percentage (android fat grams standardized to total mass of DXA android region) with significance threshold *p* < 0.01. ^2.^ Study screening question.

**Table 7:** Associations between dietary factors and android region fat percentage in men.

Diet Component (n = 47)	Estimated Coefficient	*p*-Value ^1^
Vegetable Protein (g/kg)	−40.6456	0.000028
Carbohydrate (g/kg)	−5.4602	0.000140
Phytic Acid (mg)	−0.0137	0.000316
Vegetable Protein (g/1000 kcal)	−89.8589	0.000316
Whole grains (oz)	−3.2931	0.000660
Total Dietary Fiber (g)	−0.5228	0.001071
RRR(D)-α-Tocopherol (mg)	−0.9990	0.002194
Insoluble Dietary Fiber (g)	−0.7243	0.002255
cis-9, trans-11 CLA (g)	108.2694	0.003059
Total CLA (g)	86.6468	0.003729
Vegetable Protein (g)	−0.3622	0.004224
Total Vitamin E (mg)	−0.7742	0.006298
Animal Protein (g)	0.3465	0.006371
Pectins (g)	−2.3549	0.007956
Whole Grains (oz/1000 kcal)	−5.4205	0.008147

^1.^ Univariate regression models were run to identify dietary factors associated with DXA Android fat percentage (android fat grams standardized to total mass of DXA android region) with significance threshold *p* < 0.01.

**Table 8:** Alcohol Intake is Associated with Higher Insulin Sensitivity in Women.

Parameter ^1^	Model 1	Model 2	Model 3	Model 4
	β (*p*-Value)	β (*p*-Value)	β (*p*-Value)	β (*p*-Value)
Intercept	5.997 (<0.001)	16.950 (<0.001)	5.973 (<0.001)	16.981 (<0.001)
Alcohol	3.125 (<0.001)	2.113 (0.003)	3.092 (<0.001)	2.137 (0.006)
Salty Condiments	−1.037 (0.140)	−1.249 (0.048)	−1.037 (0.144)	−1.250 (0.051)
Alcohol × Salty Condiments	−2.581 (0.004)	−1.676 (0.040)	−2.571 (0.005)	−1.682 (0.042)
Baseline BMI	--	−0.417 (<0.001)	--	−0.418 (<0.001)
Total Exercise (min/week)	--	--	0.0003 (0.921)	−0.0002 (0.933)

^1.^ Food-group (absolute portions) predictors were selected separately by sex using the feasible solutions algorithm (FSA) [20], which searches random variable pairs and their two-way interaction to maximize R^2^; the top interaction for each outcome was then tested in four ANOVA models (food group interaction only in model 1; +baseline BMI in model 2; +weekly exercise minutes in model 3; +both covariates in model 4).

**Table 9:** Whole Plant Food Groups are Associated with Higher Insulin Sensitivity in Men.

Parameter ^1^	Model 1	Model 2	Model 3	Model 4
	β (*p*-Value)	β (*p*-Value)	β (*p*-Value)	β (*p*-Value)
Intercept	3.492 (<0.001)	11.691 (<0.001)	3.156 (<0.001)	10.754 (<0.001)
Whole Grains	0.146 (0.577)	0.037 (0.872)	0.140 (0.579)	0.042 (0.853)
Baseline Nuts and Seeds	−0.466 (0.079)	−0.445 (0.059)	−0.546 (0.037)	−0.0506 (0.032)
Whole Grains × Nuts and Seeds	0.261 (0.002)	0.239 (0.002)	0.259 (0.002)	0.240 (0.002)
Baseline BMI	--	−0.294 (0.002)	--	−0.269 (0.003)
Total Exercise (min/week)	--	--	0.0004 (0.058)	0.003 (0.127)

^1.^ Food-group (absolute portions) predictors were selected separately by sex using the feasible solutions algorithm (FSA) [20], which searches random variable pairs and their two-way interaction to maximize R^2^; the top interaction for each outcome was then tested in four ANOVA models (food group interaction only in model 1; +baseline BMI in model 2; +weekly exercise minutes in model 3; +both covariates in model 4).

## Data Availability

Study data is available from the corresponding author upon reasonable request.

## Supplementary Materials

The following supporting information can be downloaded at: https://www.mdpi.com/article/10.3390/nu17213460/s1, Supplemental File S1: Merged NDSR Food Groups; Supplemental File S2: Partial Correlations; Supplemental Table S1: Past Medical History Among Participants; Supplemental Table S2: Medication Use at Baseline.

## Abbreviations

The following abbreviations are used in this manuscript:

mTOR	Mammalian/Mechanistic Target of Rapamycin
Mat-ISI	Matsuda Insulin Sensitivity Index
HOMA-IR	Homeostatic Model Assessment of Insulin Resistance
DXA	Dual-Energy X-ray Absorptiometry
CT	Computed Tomography
