# Supplementary material for: Sex Differences in Associations Between Diet and Metabolic Health in Older Adults: The Roles of Vegetable Protein and Alcohol Intake
Source: Nutrients. 2025 Nov 2;17(21):3460. doi: 10.3390/nu17213460 (PMC12608314; doi:10.3390/nu17213460)
Supplement: Supplementary file 1 [file nutrients-17-03460-s001.zip › Supplemental Tables.pdf]

Supplemental Table S1. Past Medical History Among Participants

Health Condition	Participants with Condition
Hypertension	35
Hyperlipidemia	33
History of Cancer Treatment	23
Arthritis	20
Thyroid Condition	18
Intestinal Condition	15
Cholelithiasis	8
Cardiovascular Disease	7
Peptic Ulcers	4
Gout	4
Liver Disease	3
Lung/Respiratory Disease	2
Blood Vessel Condition	2
Kidney Disease	2
Stroke	1
Seizures	1
Diabetes	0

Supplemental Table S2. Medication Use at Baseline

Medication Class	Number of Participants Taking Medications
Antidepressants/Anxiolytics	21
Blood Pressure Agents	18
Cholesterol-lowering Agents	26
Diabetes Agents	0
Diuretics	9
GERD/Acid Reflux	15
Hormones	7
Miscellaneous/Other	48
Pain/Arthritis/Anti-inflammatory	14
Thyroid Agents	17
Vitamins/Supplements	58
